# Dietary resveratrol improves immunity and antioxidant defense in ewes by regulating the rumen microbiome and metabolome across different reproductive stages

**DOI:** 10.3389/fimmu.2024.1462805

**Published:** 2024-10-11

**Authors:** Xiongxiong Li, Yuzhu Sha, Shuyan Li, Zhengwen Wang, Yanan Yang, Ting Jiao, Shengguo Zhao

**Affiliations:** ^1^ College of Animal Science and Technology, Gansu Agricultural University, Lanzhou, China; ^2^ Major in Pratacultural Science of Gansu Agricultural University, Key Laboratory of Grass Ecosystem, Ministry of Education, Sino–US Grassland Animal Husbandry Sustainable Development Research Center, Lanzhou, China

**Keywords:** Resveratrol, ewes reproduction, rumen microbiota, metabolites, immunity, antioxidant status

## Abstract

**Introduction:**

Resveratrol (Res), a natural plant antitoxin polyphenol, is widely used in animal husbandry due to its antioxidant and anti-inflammatory properties, and current research has focused on humans, sows, and female mice. This study aimed to analyze the effects of dietary Res supplementation in ewes on antioxidant activity, immune responses, hormone levels, rumen microbiota and metabolites across various reproductive stages (estrus, pregnancy, and lactation).

**Methods:**

Twenty-four healthy ewe lambs (Hu sheep, 2 months old) with a similar body weight (BW) (mean: 21.79 ± 2.09 kg) were selected and randomly divided into two groups: the control group (Con) and the Res group (Res). The Res group received 10 mg/kg Res (based on BW) in addition to their basal diet.

**Results:**

Res increased the levels of follicle-stimulating hormone (FSH), luteinizing hormone (LH), and estradiol (E2) in ewes at sexual maturity (p < 0.05). Additionally, Res supplementation induced significant increases in serum glutathione peroxidase (GSH-Px), IgG, FSH, and LH levels during estrus (p < 0.05); serum IgA, IgG and IgM during pregnancy and lactation (p < 0.05); and serum LH, glucose, GSH-Px, and catalase (CAT) levels during lactation (p < 0.05). Meanwhile, serum interleukin 1β (IL-1β) (p =0.005) and cholesterol levels (p = 0.041) during the lactation stage decreased following Res supplementation. Notably, colostrum IgA, IgG, and fat concentrations were significantly higher in the Res group than in the Con group (p < 0.05). Moreover, Res altered the rumen microbiota in ewes. Specifically, the relative abundance of Prevotella (p < 0.05) during pregnancy and Rikenellaceae_RC9_gut_group (p < 0.001) during lactation were significantly increased in ewes under Res treatment. The abundance of Rikenellaceae_RC9_gut_group was positively correlated with the levels of Ig A, Ig M, E2, FSH, LH, GSH-PX, and CAT. Additionally, Res altered the activity of metabolic pathways such as progesterone-mediated oocyte maturation, the estrogen signaling pathway, ovarian steroidogenesis, and the AMPK signaling pathway, and the levels of AICAR and 2-hydroxyestradiol metabolites, both during pregnancy and lactation.

**Discussion:**

There findings show that Res can improve health, antioxidant status, and immune activity throughout the reproductive cycle in ewes by regulating rumen microorganisms and metabolites.

## 1 Introduction

With rapid population growth, major changes have occurred in livestock production systems in recent years, especially sheep farming, where the demand for high-quality mutton is increasing, at the same time increasing the establishment of higher capacity production systems and the tendency towards integrated production activities (1). In this context, sheep health status has become one of the focuses, and ewes fecundity directly affects sheep population and farm economic benefits. Currently, improving the feeding environment, adjusting dietary nutrition, exogenous hormones to induce estrus in ewes (2), antioxidants to improve sperm kinematic parameters of spermatozoa (3) and exogenous feed additives (4) are the main means of improving the reproductive performance of ewes, which is of great importance for the development of sheep farming. The balance of the gastrointestinal microbiota in animals is directly related to host health and production performance. When animals experience changes in their external environment, diet, or other stressors, gastrointestinal dysbiosis occurs, often leading to physiological alterations (5). During pregnancy, lambing, and lactation, ewes experience physiological changes such as an increase in their nutritional requirements and violent fluctuations in hormone secretion, which may result in insufficient nutrient intake, metabolic disorders, and impaired immunity (6), all of which are exacerbated by intensive farming practices. Eventually, these changes lead to gastrointestinal dysbiosis (7). Therefore, it is vital to improve health in breeding ewes and maintain the stability of the gastrointestinal microbiota. At present, given the widespread banning of antibiotics, the use of green feed additives to alleviate oxidative stress and improve immunity and gastrointestinal microbiome balance has emerged as a hotspot of research (8). Therefore, to achieve microbiota maintenance through the use of feed additives, the effects of these additives in alleviating oxidative stress and enhancing immune function in animals are increasingly being explored.

Resveratrol (Res), a natural polyphenol (plant antitoxin) with strong biological activity (9, 10), is found in peanuts, grapes, pineapples and blueberries, among other plants (11, 12). Recent research has shown that Res can reduce oxidative stress and inflammatory responses (13–16), decrease serum lipids levels (17, 18), and improve lipid metabolism in animals (19, 20). The anti-inflammatory and antioxidant effects of Res have been linked to a reduction in the levels of inflammatory factors, such as tumor necrosis factor (TNF)-α, interleukin (IL)-1β, and interleukin (IL)-6 (21), and an increase in the activities of catalase (CAT), superoxide dismutase (SOD), and glutathione peroxidase (GSH-Px) activities (22, 23). Furthermore, the chemical structure of Res is similar to that of some estrogens, such as diethylstilbestrol (DES), and it is thus considered a natural phytoestrogen and therefore a modulator of female reproductive function (24). Previous studies indicate that Res can improve reproductive performance in sows and female mice, thereby improving offspring health (25–29). Additionally, Res has been found to mitigate oxidative stress by regulating the intestinal microbiota and metabolome of piglets (8) and by inhibiting methane production and improving the rumen microbiota of sheep (30). However, the effects of Res on ewes have so far not been reported. We hypothesized that Res could improve antioxidant activity, immunity, and hormone levels in breeding ewes by regulating the rumen microbiota and metabolites and thereby improving ewe health.

To test this hypothesis, we examined the effects of continuous dietary Res supplementation on immunity and antioxidant performance in ewes throughout various reproductive stages, i.e., sexual maturity, estrus, pregnancy, and lactation, and also examined changes in rumen microbes and metabolites at each stage. The primary aim of this study was to understand whether Res enhances the immunity and antioxidant capacity of ewes by regulating the rumen microbiome and metabolites.

## 2 Materials and methods

### 2.1 Materials

Res (99% purity) was purchased from Henan Shenghao Biotechnology Co., LTD., China. It is white to pale yellow powder, insoluble in water, easily soluble in organic solvents such as ethanol, no special odor. The specific extraction process is as follows: take polygonum cuspidatum sieb as the raw material, through the process of fermentation-extraction-concentration-dissolution- separation-filtration-concentration-separation, finally get resveratrol powder.

### 2.2 Experimental animals

Twenty-four weaned ewe lambs (Hu sheep) were obtained from a well-managed and large-scale farm in Linxia, Gansu Province, China, with similar body condition, good health status, and an average weight of 21.79 ± 2.09 kg. Before the test began, all the lambs underwent deworming, brucellosis testing, and vaccination to ensure that all experimental animals were healthy and disease-free. All animal protocols were approved by the Animal Committee of Gansu Agricultural University (Approval No. GSAU-Eth-AST-2023-035).

### 2.3 Experimental design and sample collection

#### 2.3.1 Experimental design and feeding management

**Figure 1:**
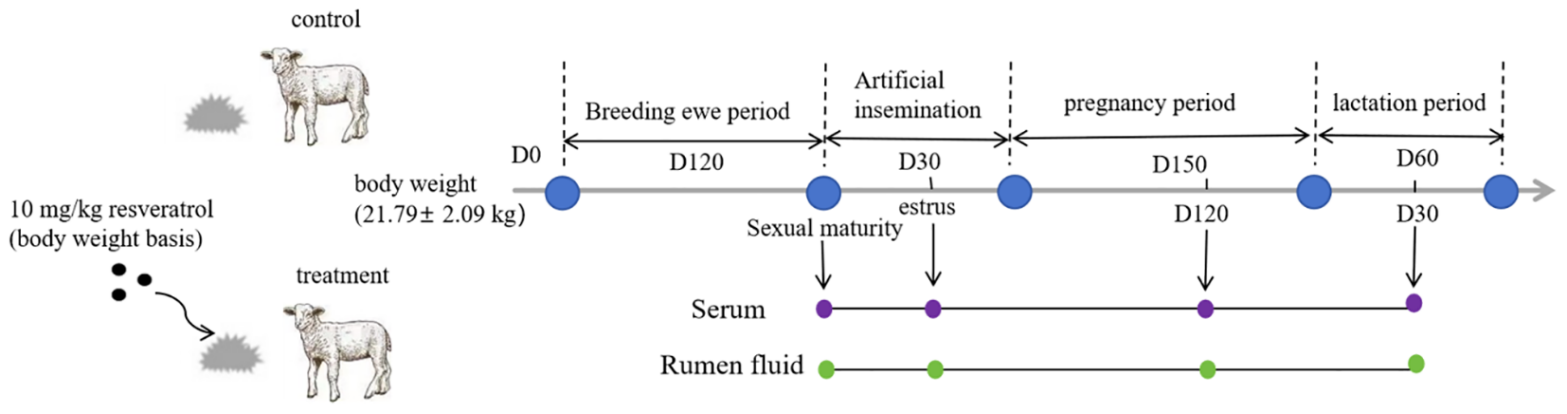
Experimental design.

Twenty-four healthy ewe lambs (2 months old) with an average body weight (BW) of 21.79 ± 2.09 kg were selected for the study and randomly divided into two groups: the control group (Con) and the resveratrol group (Res) (n = 12). The lambs in the Con group were fed a basal diet, while the lambs in the Res group received an additional 10 mg/kg of Res (based on BW). Res was weighed and mixed evenly in the basal diet evenly before feeding. The amount of Res to be added was based on the findings reported by Wang et al. (31) and Zhang (32) et al. The experimental design is shown in **Figure 1**. During the experiment period, all ewes were fed in Jinheyuan Agricultural Farm in Gansu Province, China. Ewes were fed TMR feed twice daily (08:00 and 18:00), and they had *ad libitum* access to fresh tap water. The ewes’ experimental diets were formulated according to the Agricultural Industry Standard Mutton Sheep Feeding Standard of the People’s Republic of China. The dietary composition and nutrient content are shown in **Supplementary Table S1**.

#### 2.3.2 Estrus synchronization and artificial insemination

All ewes were fed until sexual maturity (7 months of age) and subjected to estrus synchronization and artificial insemination thereafter (8 months old). First, one Shengyuan Sheep Vaginal Sponges (Henan University of Animal Husbandry and Economy, Zhengzhou, China) was placed into each sheep vagina, and 10 days later, each ewe was injected with 500 IU of Serum Gonadotroph for Injection. The Shengyuan Sheep Vaginal Sponges was removed after 11 days, when the ewe was in estrus 2 days later, 1 mL of semen from rams (Hu sheep) was collected and diluted 10 times for intra-vaginal insemination, and then repeated again after 24 h. Activity was tested by allowing a virile ram wearing an isolate cloth to show complete sexual behavior to the ewe at 08:00 and 17:00 every day. Ewes that accepted the crawling behavior of the ram were considered to be in estrous. Pregnancy was detected in ewes using B-ultrasound 45 days after artificial insemination.

#### 2.3.3 Sample collection

Six ewes were randomly selected from each group to collect blood and rumen fluid were collected at estrus and during pregnancy (120 d of gestation) and lactation (30 d after delivery). Blood samples were collected via the jugular vein and centrifuged at 3500 rpm for 10 min to obtain serum. The serum samples were stored at −80 °C for the subsequent evaluation of serum hormone levels, biochemical indexes, antioxidant indexes, and immune indexes. Rumen fluid samples were collected through oral cavity using a rumen tube. Briefly, a flexible PVC tube with holes of 2.5 mm diameter in the 15 cm-probe head (Anscitech Co., Ltd., Wuhan, China) was connected to an electric vacuum pump (7 mbar) and inserted into the ewes rumen via the esophagus to collect the rumen sample. About 60 mL of rumen fluid from each ewe was collected 3 h after morning feeding. The first part was placed in a 50 mL centrifuge tube and stored at −20°C for the subsequent determination of fermentation parameters. The second part was placed in a 5 mL cryotube and stored at −80°C for rumen microbiome and metabolome analysis. Colostrum was also collected from the ewes within 2 days of lambing to determine immune indexes and milk quality, and the birth weight and growth of the lambs were measured and monitored until weaning (60 d).

### 2.4 Measurement indexes and method

#### 2.4.1 Serum biochemical indexes and antioxidant enzyme activity

The levels of serum aspartate aminotransferase (AST), blood urea nitrogen (BUN), cholesterol (CHO), alanine aminotransferase (ALT), high-density lipoprotein (HDL), triglyceride (TG), total protein, (TP) and glucose (GLU) were measured using a Mindray BS-230 Automatic Biochemical Analyzer (Shenzhen, China) (33). The antioxidant enzyme activity of serum total superoxide dismutase (T-SOD), glutathione peroxidase (GSH-Px), malondialdehyde (MDA), total antioxidant capacity (T-AOC) and catalase (CAT) were determined using kits (Nanjing Jiancheng Bioengineering Institute, Nanjing, China). The kits used were as follows: A001-1-2, A005-1-2, A003-1-2, A015-2-1, and A007-1-1.

#### 2.4.2 Sex hormones and immune indexes measurements

The sex hormones and immune indexes of serum follicle-stimulating hormone (FSH), luteinizing hormone (LH), progesterone (P4), estradiol (E2), colostrum immunoglobulin A (IgA), immunoglobulin G (IgG), immunoglobulin M (IgM), interleukin 1β (IL-1β), and TNF-α were determined using commercial ELISA kits (Shanghai Enzyme-Linked Biotechnology Co., Ltd., Shanghai, China) according to the manufacturer’s instructions. The kits used were as follows: F3906-A, F3921-A, F3911-A, F3907-A, F72006-A, F3902-A, F72008-A, F3895-A, and F72110-A.

#### 2.4.3 Determination of SCFAs, ammonia nitrogen, and milk quality

pH values were recorded using a pH meter (P611, Shanghai, China) and a glass electrode immediately after rumen fluid extraction. The ruminal NH_3_-N concentrations were measured via colorimetric methods using a 721-type spectrophotometer (TU-1901). Volatile fatty acids (VFAs) were identified using gas chromatography (GC-2010 Plus; Shimadzu, Kyoto, Japan) (34). The levels of milk protein, lactose, non-fat solids, and milk fat in ewe colostrum were determined with MilkoScan FT1.

#### 2.4.4 DNA extraction and sequencing

DNA was extracted from rumen fluid using a bacterial DNA extraction kit (Omega, Shanghai, China). DNA concentration and purity were determined using a NanoDrop 2000 UV-VIS spectrophotometer (Thermo Scientific, Wilmington, USA). Then, the V3-V4 hypervariable region of the bacterial 16S rRNA gene was amplified using the primer pairs 338F: 5’- ACTCCTACGGGAGGCAGCA-3’ and 806R: 5’- GGACTACHVGGGTWTCTAAT-3’. PCR amplification products were purified with an Omega DNA Purification Kit (Omega Inc., Norcross, GA, USA), and paired-end (2 × 250 bp) sequencing was performed on the Illumina Nnovaseq600 platform. Raw sequences were subsequently filtered using an in-house program.

Clean reads were subjected to feature classification to identify amplicon sequence variants (ASVs), and the ASVs conuts less than 2 in all samples were filtered, Then, species annotation was performed by comparing the ASVs to the sequences in the SILVA (Release 138.1) database at a confidence threshold of 70% (35) to analyze microbial community structure and species clustering. The alpha analysis of each sample was examined using QIIME2 software, and beta diversity calculations were performed using principal coordinate analysis (PCoA). The differences between groups were tested using Anosim and Adonis examine whether these differences were significantly greater than intragroup differences. Bacteria with LDA scores >2.5 were considered to show differential abundance between the Con and Res groups. Subsequently, t-tests (Metastats software) were used to compare the species abundance data between groups, and screened the different bacterial genera between the two groups.

#### 2.4.5 Metabolomics analysis of rumen fluid

Metabolomics analysis of the rumen fluid was performed using liquid chromatography coupled with mass spectrometry (LC-MS). First, the metabolites in rumen fluid were extracted as follows: 100 μL samples were weighed after thawing at room temperature, and 500 μL extraction solution containing internal standard (methanol: acetonitrile v/v = 1:1, 2-chloro-L-phenylalanine, 20 mg/L) was added. The solution was vortexed and mixed for 30 s, sonicated for 10 min (ice water bath), and incubated at −20°C for 1 h. Then, the mixture was centrifuged for 15 min (4°C, 12000 rpm). The extract was dried and concentrated after removing 500 μL of the supernatant, and 160  μL of acetonitrile and water were added in a 1:1 ratio. The above steps were repeated twice. Tandem extraction was performed on a Waters Xevo Acquity I Class PLUS ultra-high performance liquid chromatograph (UPLC) and a Waters Xevo G2-XS QTOF High-resolution Mass Spectrometer (HRMS). A Waters Xevo Acquity UPLC HSS T3 column (1.8 um; 2.1*100 mm) was used. Progenesis QI software was used for peak extraction and peak comparison when analyzing metabolome data, and metabolites were identified based on the METLIN database and public databases. The theoretical fragment identification deviation and mass deviation were within 100 ppm. The repeatability of within group results and quantity control sample results were determined using principal component analysis (PCA) and Spearman’s correlation analysis. Additionally, BMKCloud was used to conduct a subsequent bioinformatics analysis of the identified metabolites. Finally, the differential metabolites were screened by combining the differential multiple in the OPLS-DA model, t-test *P* value and VIP value, and the screening standard was *P* value < 0.05 and VIP > 1. Moreover, KEGG pathway enrichment analysis was performed for differential metabolites (36).

### 2.5 Correlation and statistical analysis

The independent sample t-test in IBM SPSS Statistics 22 software was used to compare the hormone levels, antioxidant activity, immune indexes, and fermentation characteristics between the two groups during each reproduction period. Spearman’s correlation test was employed to calculate the correlation among serum parameters, rumen microbes (Top20), and differential metabolites, and the same method was used for examining correlations between differential metabolites and rumen microbes (Top20). The correlation coefficients (r) ranged from −1 to 1. r > 0 and < 0 represented positive and negative correlations, respectively. The |r| value denoted the degree of correlation among variables. In particular, r = −1, 0, and 1 reflected a completely negative correlation, nocorrelation and a completely positive correlation, respectively.

## 3 Results

### 3.1 Effect of Res on body weight and hormone levels in ewes at sexual maturity

**Figure 2:**
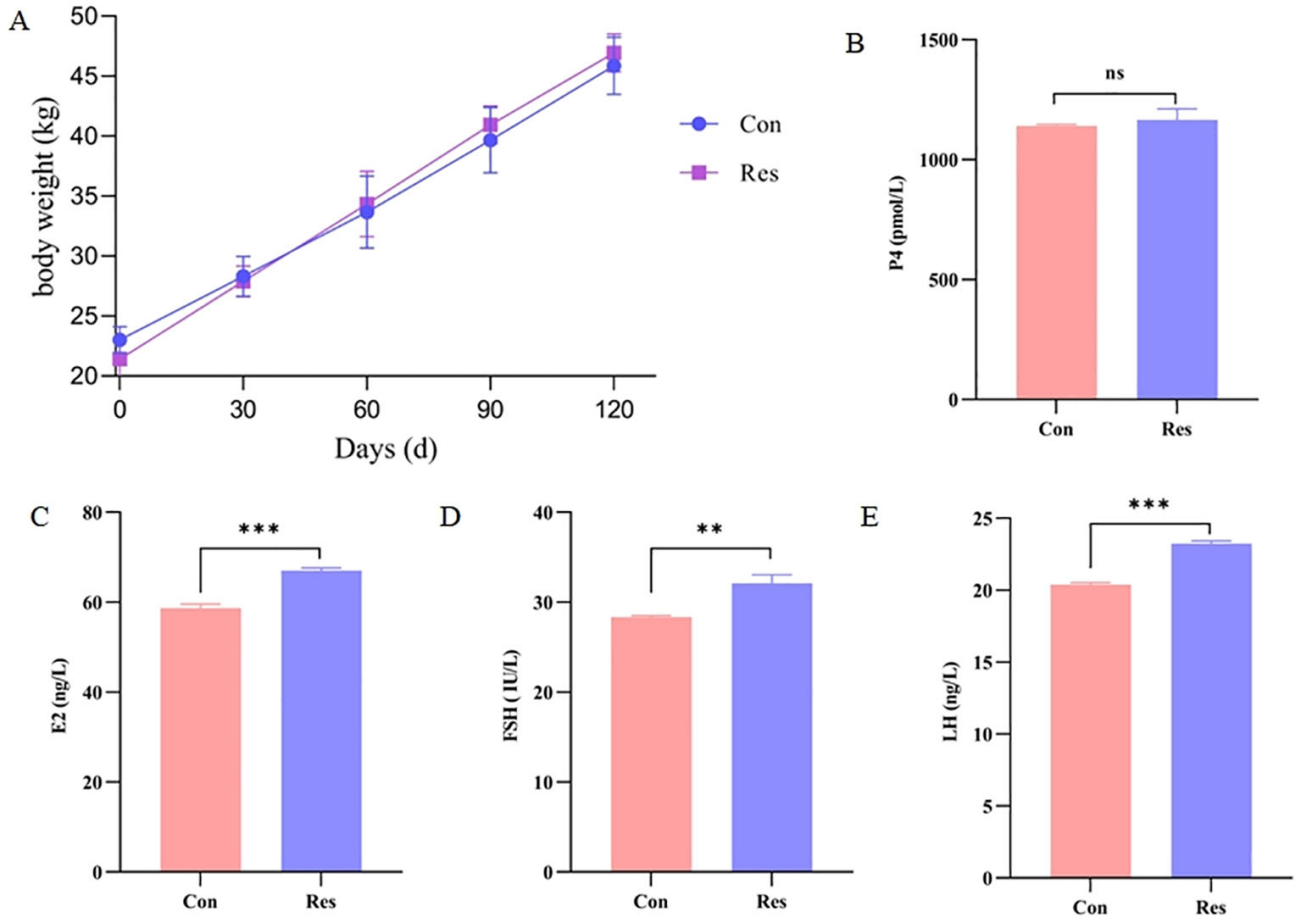
Dietary Res supplementation had no significant effect on the body weight of ewes at sexual maturity (p > 0.05) **(A)**. However, it significantly increased the levels of the hormones estradiol (E2) (67.05 ng/L), follicle-stimulating hormone (FSH) (32.13 IU/L), and luteinizing hormone (LH) (23.24 ng/L) (p < 0.01), but no significant effect (p > 0.05) on progesterone (P4) **(B–E)**. **P < 0.01, ***P < 0.001.

Dietary Res supplementation had no significant effect on the body weight of ewes at sexual maturity (*p* > 0.05) (**Figure 2A**). However, it significantly increased the levels of the hormones estradiol (E2) (67.05 ng/L), follicle-stimulating hormone (FSH) (32.13 IU/L), and luteinizing hormone (LH) (23.24 ng/L) (*p* < 0.01).

### 3.2 Effect of Res supplementation on serum indexes

**Figure 3:**
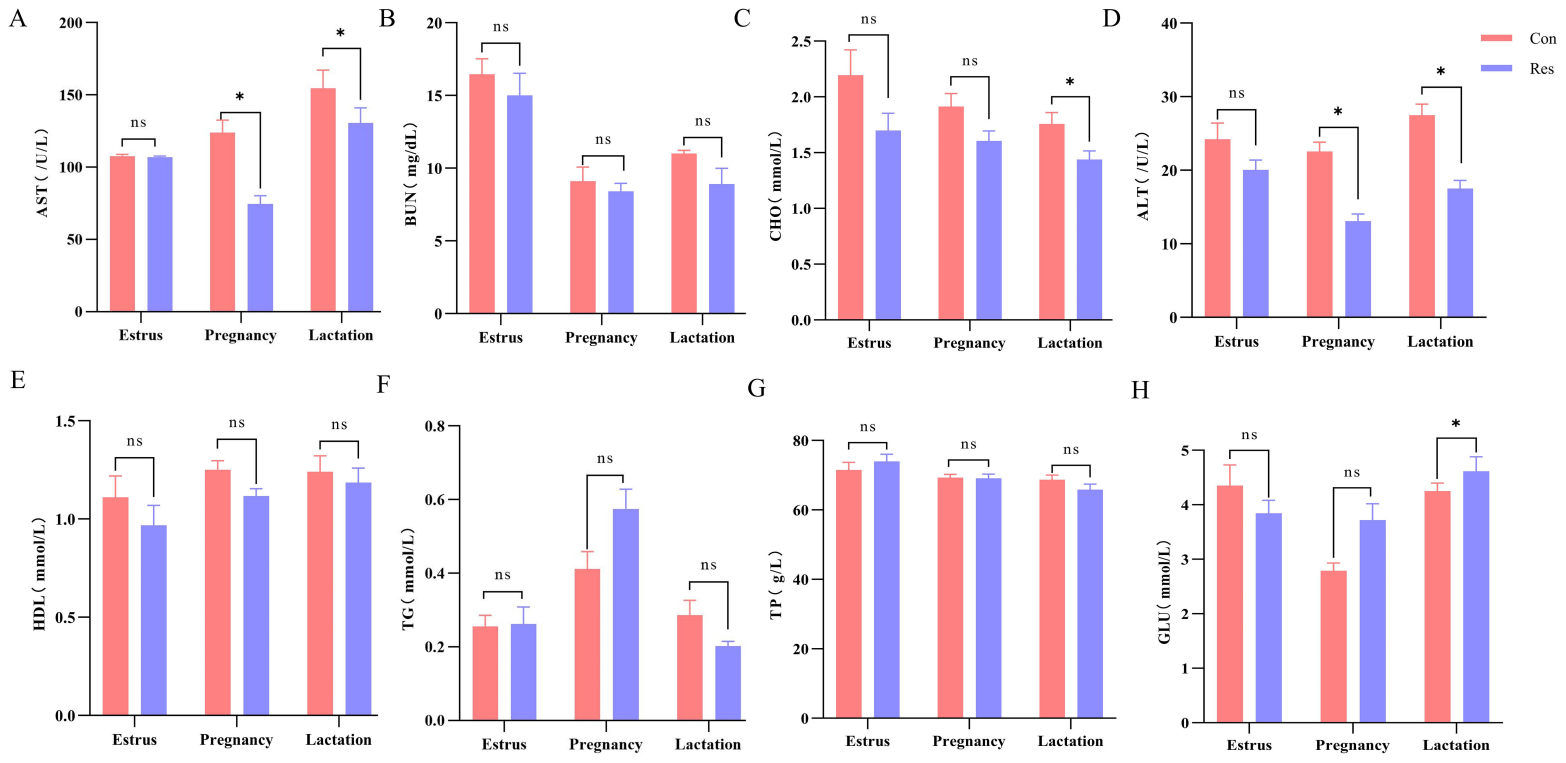
Effect of dietary Res supplementation on the serum indexes of ewes at different reproductive stages. Con, control; Res, Resveratrol; AST, aspartate aminotransferase; BUN, blood urea nitrogen; CHO, cholesterol; ALT, alanine aminotransferase; HDL, high-density lipoprotein; TG, triglyceride; TP, total protein; GLU, glucose. All values are expressed as means ± SEM (n = 6). *P < 0.05.

The aspartate aminotransferase (AST) (74.62 U/L, pregnancy; 137.86 U/L, lactation) and alanine aminotransferase (ALT) (13.07 U/L, pregnancy; 17.50 U/L, lactation) concentrations were significantly lower (*p* < 0.05) in the Res group during pregnancy and lactation. Meanwhile, during lactation, cholesterol (CHO) levels were lower (*p* = 0.041) in the Res group (1.44 mmol/L) than in the Con group (1.80 mmol/L), while glucose (GLU) levels were higher (*p* = 0.039) in the Res group (4.61 mmol/L) than in the Con group (4.00 mmol/L). There were no significant differences in blood urea nitrogen (BUN), high-density lipoprotein (HDL), triglyceride (TG), and total protein (TP) levels between the Res and Con groups (*p* > 0.05) (**Figure 3**).

### 3.3 Effects of Res supplementation on antioxidant activity, immune responses and hormone levels

**Figure 4:**
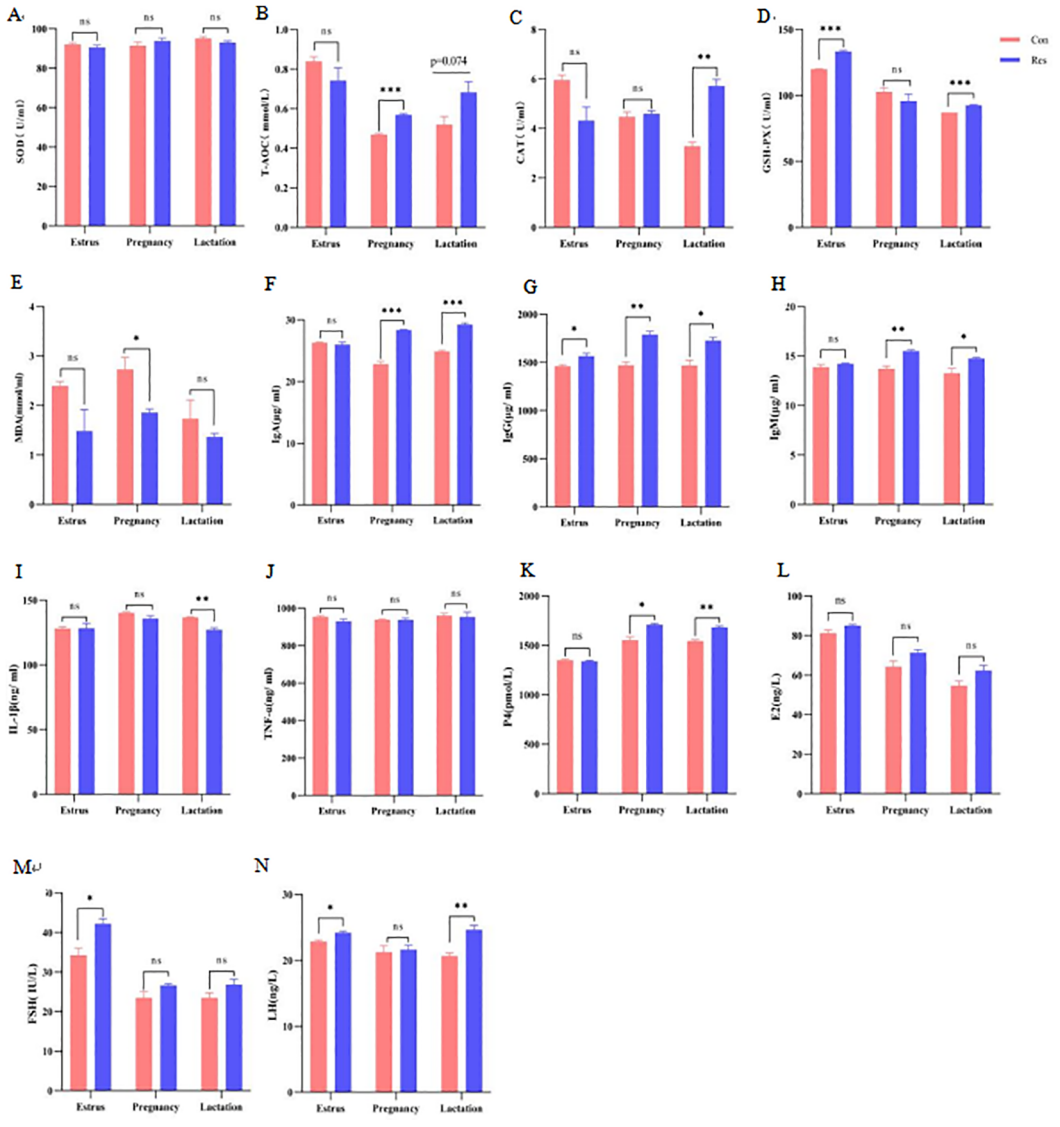
Effect of dietary Res supplementation on the antioxidant status, immune activity and hormone levels of ewes. Con, control; Res, Resveratrol. **(A–E)** antioxidant index. SOD, superoxide dismutase; T-AOC, total antioxidant capacity; GSH-Px, glutathione peroxidase; CAT, catalase; MDA, malondialdehyde. **(F–J)** Immune index. IgA, Immunoglobulin A; IgG, immunoglobulin G; IgM, immunoglobulin M; IL-1β, interleukin 1β; TNF-α, tumour necrosis factor α. **(K–N)** Reproductive hormone. E2, estradiol; P4, progesterone; FSH, follicle-stimulating hormone; LH, luteinizing hormone. All values are expressed as means ± SEM (n = 6). *P < 0.05, **P < 0.01, ***P < 0.001.

The activity of serum glutathione peroxidase (GSH-Px) (133.38 U/mL, estrus; 92.71 U/mL, lactation) increased significantly during the estrus and lactation stages following Res treatment (*p =*0.031). Meanwhile, the Res group showed increased total antioxidant capacity (T-AOC) (0.57 mmol/L) during pregnancy (*p* < 0.001) and increased catalase (CAT) activity (5.72 U/mL) during lactation (*p =* 0.001), as well as decreased malondialdehyde (MDA) activity (1.86 mmol/mL) during pregnancy (*p* = 0.027). The concentrations of serum IgA (28.37 μg/mL, pregnancy; 29.24 μg/mL, lactation), IgG (1788.66 μg/mL, pregnancy; 1727.39 μg/mL, lactation), and IgM (15.51μg/mL, pregnancy; 14.75 μg/mL, lactation) were significantly higher (*p* < 0.05) in the Res group than in the Con group during pregnancy and lactation, and the IgG concentrations (1565.65 μg/mL) were higher even at the estrus stage in ewes receiving Res (*p* = 0.031). However, Res showed no significant effects on inflammatory factors, except decreased IL-1β (127.14, ng/mL) during the lactation stage (*p* = 0.005). Notably, progesterone (P4) (1684.64, pmol/L) and LH (24.67, ng/L) levels were significantly higher (*p* < 0.05) in the Res group than in the Con group during lactation, while FSH (42.23, IU/L) and LH (24.22, ng/L) were significantly increased in the Res group (*p* < 0.05) at the estrus stage (**Figure 4**).

### 3.4 Effects of Res supplementation on rumen fermentation characteristics

**Figure 5:**
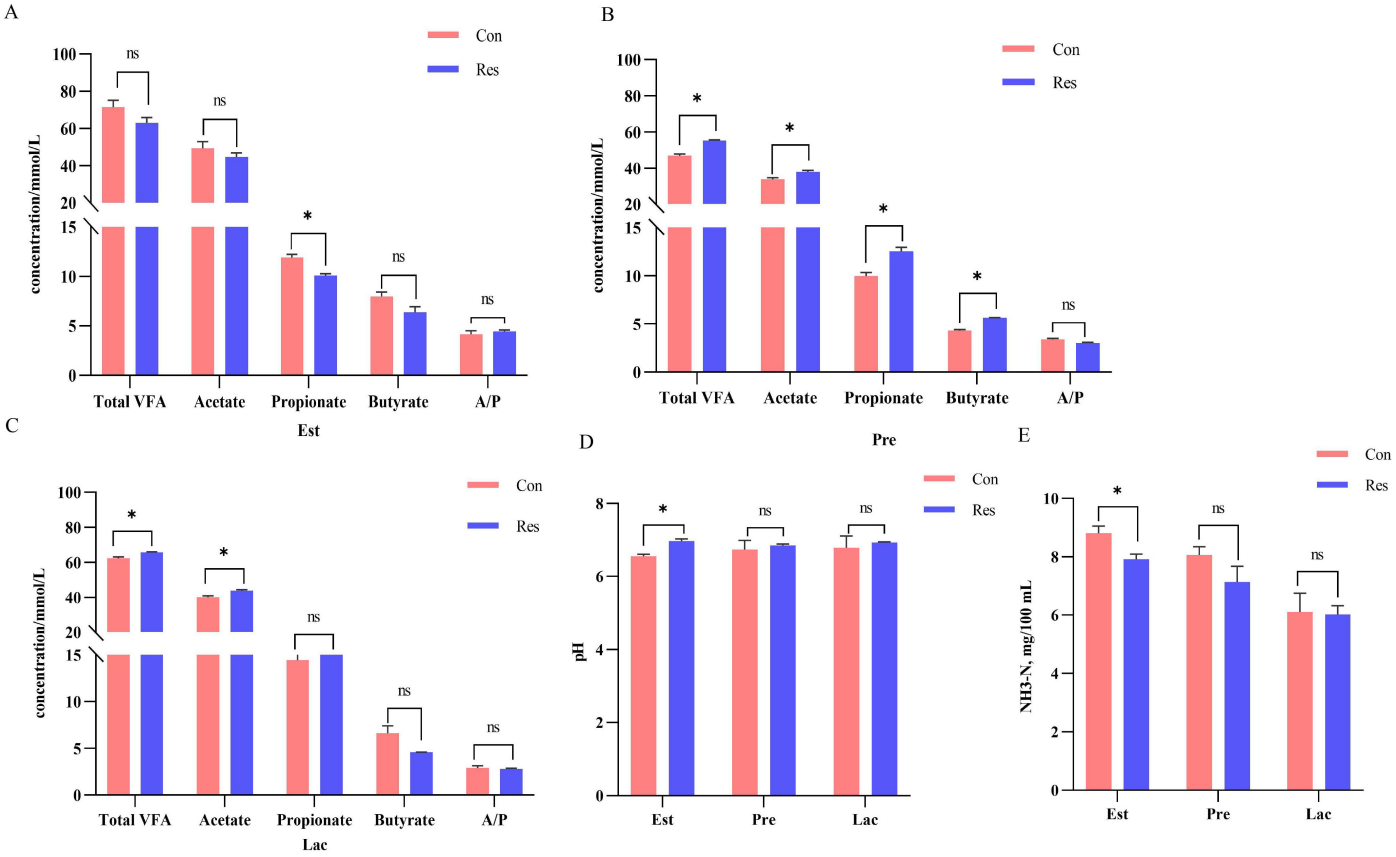
Effect of dietary Res supplementation on rumen fermentation characteristics in ewes at different reproductive stages. **(A–C)** Rumen fluid VFA during estrus, pregnancy and lactation, respectively. **(D)** pH. **(E)** NH_3_-N. Con, control; Res, Resveratrol; NH_3_-N, Ammonia nitrogen; VFA, volatile fatty acid; A/P, acetate to propionate ratio. All values are expressed as means ± SEM (n = 6). *P < 0.05.

The rumen pH value increased (*p* = 0.001) in the Res group (6.73) than in the Con group (6.55) during estrus, while the NH_3_-N (7.92 mg/100 mL, *p* = 0.016) and propionate (10.09 mmol/L, *p* = 0.007) concentrations decreased under Res treatment. Meanwhile, the total VFA (55.30 mmol/L, pregnancy; 65.81 mmol/L, lactation) and acetate (37.78 mmol/L, pregnancy; 43.90 mmol/L, lactation) concentrations increased significantly (*p* < 0.05) after Res treatment during pregnancy and lactation, whereas propionate (12.55 mmol/L, *p* = 0.011) and butyrate concentrations (5.65 mmol/L, *p* < 0.001) only increased during pregnancy (**Figure 5**).

### 3.5 Changes in ruminal bacterial communities following Res treatment

**Figure 6:**
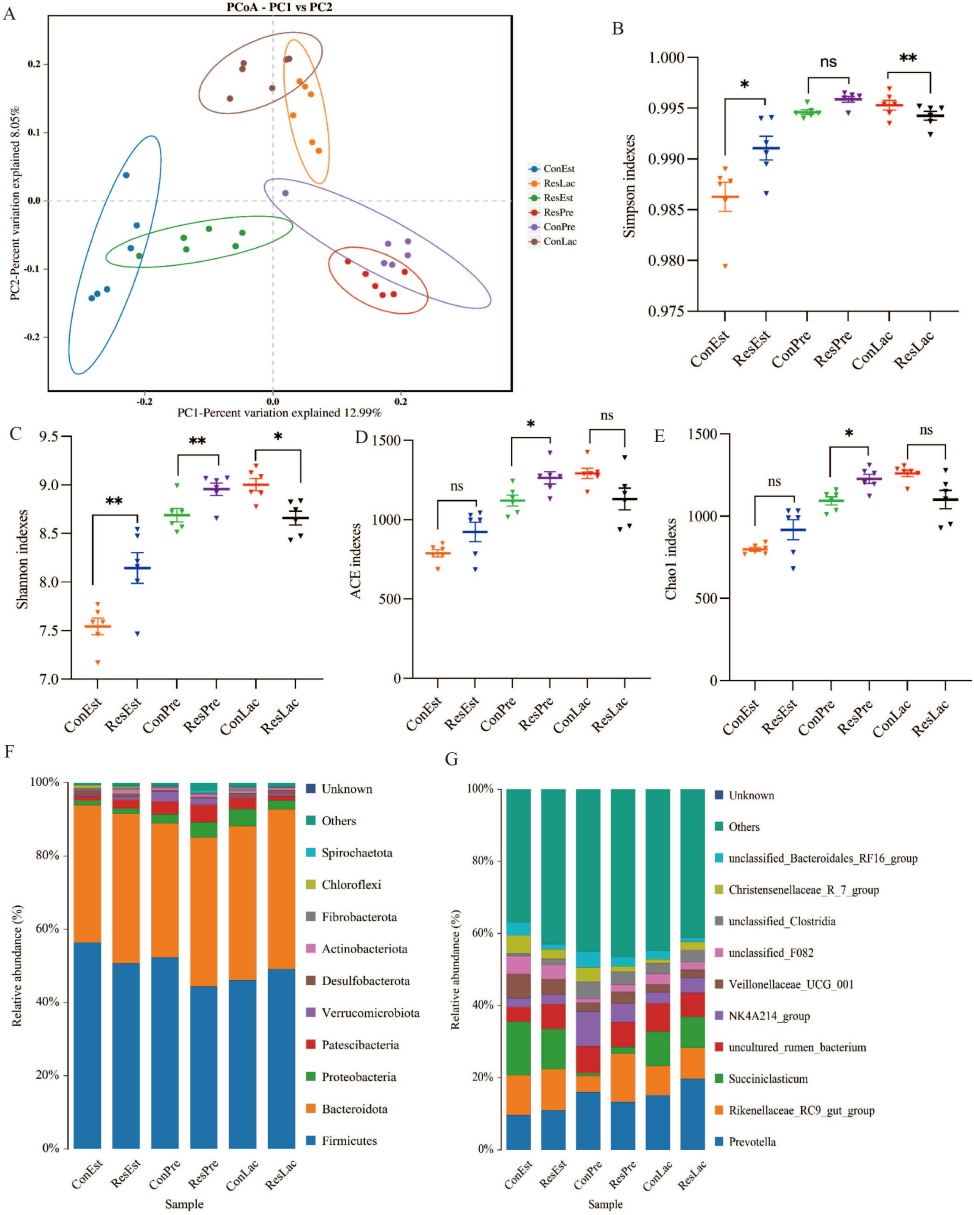
Effect of dietary Res supplementation on ruminal microbiota composition in ewes. **(A)** PCoA analysis; Alpha-diversity indices included the Simpson **(B)**, Shannon **(C)**, ACE **(D)** and Chao1 **(E)**. The relative abundances at the **(F)** phylum and **(G)** genus levels of ruminal microbiota. All values are expressed as means ± SEM, n = 6. * or ** represent a significant or extremely significant difference, *P < 0.05, **P < 0.01. ConEst, control in estrus; ResEst, resveratrol in estrus; ConPre, control in pregnancy; ResPre, resveratrol in pregnancy; ConLac, control in lactation; ResLac, resveratrol in lactation.

A total of 2,850,553 reads were obtained from 36 rumen fluid samples. Furthermore, 2,832,831 clean reads were obtained after quality control and splicing, resulting in an average value of 78,690 clean reads per sample. The top 10 phyla and genera of ruminal bacteria (in terms of relative abundance) are shown in **Figures 6F, G**. The ASV dilution curves and the species accumulation plots demonstrated that the sequencing depth obtained in this study was sufficient to characterize the bacterial microbiota of the samples (**Supplementary Figure S1**). In addition, PCoA showed that the rumen microbial community structure of the two groups was obviously different at three reproductive stages (**Figure 6A**). The α-diversity analysis revealed that the ACE (*p* = 0.019), Chao 1 (*p* = 0.019), Simpson (*p* = 0.007) and Shannon (*p* = 0.018) indexes were significantly higher in the Res group than in the Con group during lactation, while the Shannon (*p* = 0.005) index was significantly higher in the Res group during pregnancy (**Figures 6B–E**). These findings indicated that Res could increase microbial community richness and diversity in the rumen of ewes.

Next, the relative abundances of the top 10 microbes at the estrus, pregnancy, and lactation stages were analyzed. At the phylum level (**Figure 6F**; **Supplementary Table S2**), *Firmicutes* and *Bacteroidetes* were the dominant phyla in all samples, accounting for more than 88% of the total microbes. Metastats analysis showed that the relative abundance of *Bacteroidota* (41.00%, *p* = 0.013), *Patescibacteria* (2.30%, *p* = 0.033), and *Proteobacteria* (1.47%, *p* = 0.031) was significantly higher under Res treatment during estrus, while the relative abundance of *Firmicutes* (50.50%, *p* = 0.007) and *Desulfobacterota* (0.93%, *p* = 0.022) was lower. Additionally, the relative abundance of *Patescibacteria* (1.20%, *p* < 0.001) and *Proteobacteria* (2.77%, *p* = 0.008) was significantly lower under Res treatment during pregnancy. The relative abundance of *Proteobacteria* (4.44%, *p* = 0.020), *Cyanobacteria* (0.76%, *p* = 0.028) and *Spirochaetota* (0.73%, *p* = 0.048) was significantly higher in the Res group during lactation.

At the genus level (**Figure 6G**, **Supplementary Table S3**), compared to the Con, the relative abundance of *Veillonellaceae_UCG_001* (4.28%, *p* = 0.002) and *unclassified_Bacteroidales_RF16_group* (1.59%, *p* = 0.041) was significantly lower under Res treatment at the estrus stage, while *uncultured_rumen_bacterium* (6.81%, *p* = 0.070) tended to increase. During the pregnancy stage, the relative abundance of *unclassified_F082* (2.16%, *p* = 0.029), *Saccharofermentans* (1.37%, *p* < 0.001), *unclassified_Lachnospiraceae* (2.08%, *p* = 0.007), and *Prevotellaceae_UCG_001* (1.35%, *p* = 0.003) was lower under Res treatment, while that of *Prevotella* (19.40%, *p* = 0.009) was significantly higher. During the lactation stage, the relative abundance of *Rikenellaceae_RC9_gut_group* (13.13%, *p* < 0.001) was significantly higher under Res treatment, while that of *unclassified_Bacteroidales_RF16_group* (2.67%, *p* = 0.032), *Prevotellaceae_UCG_001* (1.12%, *p* = 0.010) and *Christensenellaceae_R_7_group* (1.36%, *p* = 0.003) was lower.

LEfSe difference analysis was performed to examine the rumen microorganisms identified in the two groups at different reproduction stages (**Supplementary Figure S2**). During the estrus stage, the main microbial groups in the Con group were *Succiniclasticum*, *uncultured rumen bacterium* and *Veillonellaceae_UCG_001*. During pregnancy, the main microbial groups in the Con group were *Proteobacteria*, *unclassified Rikenellaceae RC9 gut group* and *unclassified Succiniclasticum.* Meanwhile, the main microbial group in the Res group was *Prevotella*. During lactation, the main microbial groups were *NK4A214_group*, *Prevotellaceae UCG 001* and *unclassified_Bacteroidales_RF16_group* in the Con group and *Rikenellaceae RC9 gut group* and *uncultured rumen bacterium* in the Res group.

### 3.6 Effect of Res supplementation on the microbial metabolic profile of the rumen

**Figure 7:**
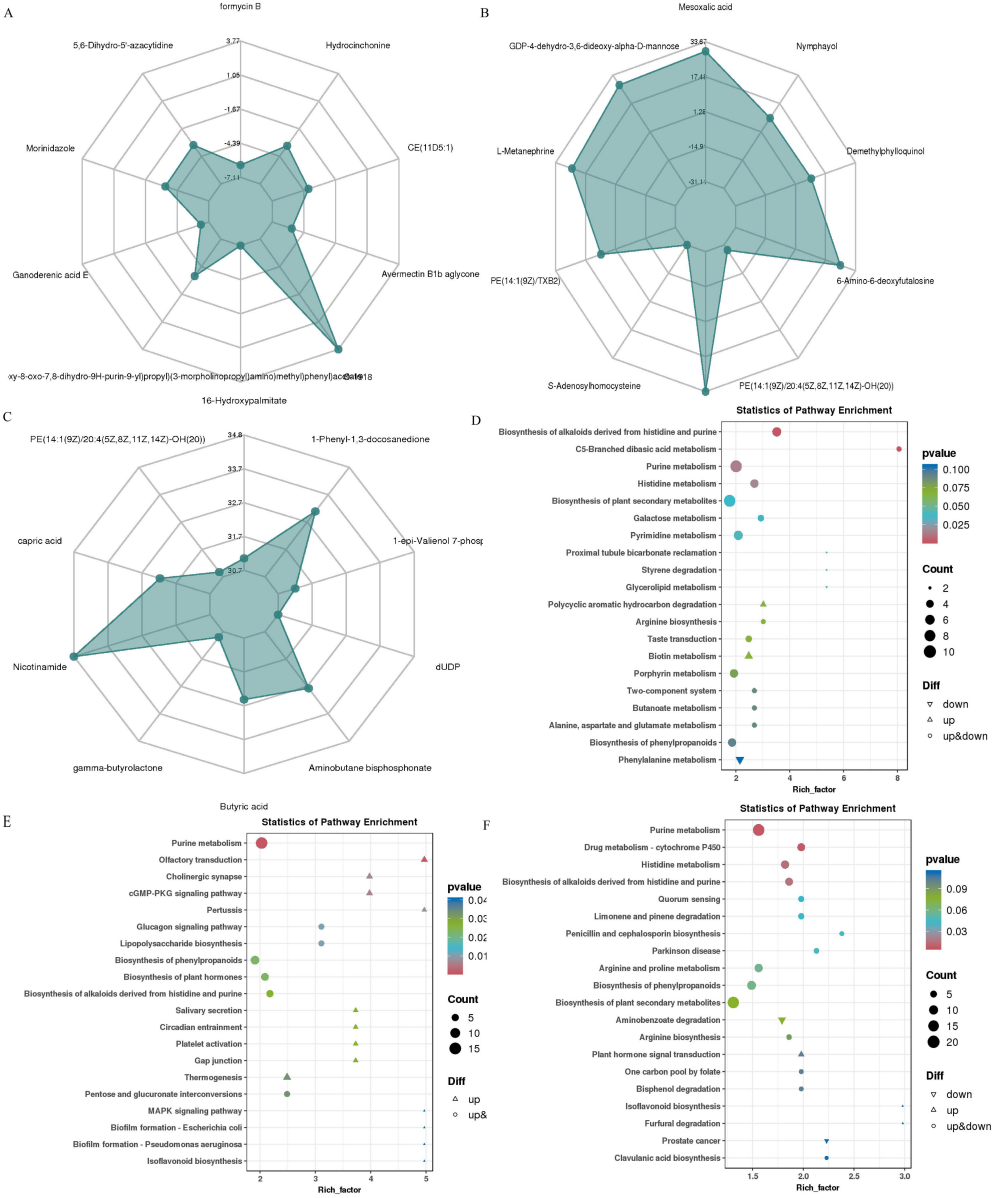
Effect of Res supplementation on ewes ruminal metabolites profile. **(A–C)** Radar distribution maps of the top 10 differential metabolites with the largest absolute log2FC values for ConEst vs ResEst, ConPre vs ResPre and ConLac vs ResLac, respectively; **(D–F)** The KEGG functional enrichment map of differential metabolites of ConEst vs ResEst, ConPre vs ResPre and ConLac vs ResLac, respectively. ConEst, control in estrus; ResEst, resveratrol in estrus; ConPre, control in pregnancy; ResPre, resveratrol in pregnancy; ConLac, control in lactation; ResLac, resveratrol in lactation.

The metabolome of 36 rumen fluid samples was qualitatively and quantitatively analyzed using an LC-QTOF platform. In total, 3,676 metabolites (positive and negative ion mode) were detected. Principal component analysis (PCA) clustering plots showed that the rumen microbial metabolic profiles of the Con and Res groups were significantly different across the three reproductive stages (**Supplementary Figure S3**). To further compare the distribution of these rumen metabolites, OPLS-DA was conducted (**Supplementary Figure S4**). The results showed distinct clustering between the Con in estrus (ConEst) vs. Res in estrus (ResEst) groups, Con in pregnancy (ConPre) vs. Res in pregnancy (ResPre) groups, and Con in lactation (ConLac) vs. Res in lactation (ResLac) groups. Based on the screening criteria FC > 1, *P* value < 0.05, and VIP > 1, 488, 711, and 1265 differential metabolites, respectively, were detected in these paired group comparisons (**Supplementary Figure S3A**, **Supplementary Table S4**). The top 10 differential metabolites with the highest absolute value of log2FC were selected for radar analysis. During the estrus stage (**Figure 7A**), differential metabolites such as 5,6-Dihydro-5’-azacytidine, Morinidazole, and Hydrocinchonine were identified. During the pregnancy stage (**Figure 7B**), differential metabolites such as L-Metanephrine, Mesoxalic acid, PE(14:1(9Z)/XB2), Ergothioneine, and 6-Amino-6-deoxyfutalosine were identified. Finally, during the lactation stage (**Figure 7C**), differential metabolites such as Nicotinamide and 1-Phenyl-1,3-docosanedione were identified. The KEEG functional enrichment analysis of the differential metabolites is illustrated in **Figures 7D–F**. The critical differential metabolic pathways identified were as follows: (i) purine metabolism, histidine metabolism, pyrimidine metabolism, porphyrin metabolism, biosynthesis of phenylpropanoids, and phenylalanine metabolism during the estrus stage; (ii) purine metabolism, glucagon signaling pathway, lipopolysaccharide biosynthesis, biosynthesis of phenylpropanoids, and pentose and glucuronate interconversions during the pregnancy stage; (iii) and purine metabolism, histidine metabolism, arginine and proline metabolism, and biosynthesis of phenylpropanoids during the lactation stage.

We further analyzed the alterations in metabolites following Res supplementation at different reproductive stages. KEGG functional annotation and enrichment analyses revealed a total of 168 differential metabolic pathways in the ResEst vs. ConEst groups (**Supplementary Table S5**). The major pathways included the AMPK signaling pathway, glucagon signaling pathway, alpha-Linolenic acid metabolism, folate biosynthesis, and biosynthesis of terpenoids and steroids. Among the metabolites associated with these pathways, AICAR, 2-Oxoglutarate, 3-Hexenal, Folate, Dihydropteroate, and Cinnamaldehyde showed higher levels in ResEst, whereas Fumaric acid, Estrone glucuronide, 2(R)-HOT, Fumaric acid, Ectoine, D-Serine, and D-erythro-3-Methylmalate showed higher levels in ConEst (**Supplementary Table S6**). Compared to ConPre, ResPre showed a total of 252 differential metabolic pathways (**Supplementary Table S5**). The key pathways included the insulin signaling pathway, oocyte meiosis, MAPK signaling pathway, progesterone-mediated oocyte maturation, cAMP signaling pathway, estrogen signaling pathway, and ovarian steroidogenesis. Of the related metabolites, cAMP, Traumatin, alpha-Linolenic acid, Trioxilin B3, Hepoxilin B3, 5-HETE, L-Leucine, L-Homophenylalanine, and Acetyl-CoA showed higher levels in ResPre (**Supplementary Table S6**). Compared to ConLac, ResLac had a total of 267 differential metabolic pathways (**Supplementary Table S5**), including oocyte meiosis, progesterone-mediated oocyte maturation, arachidonic acid metabolism, steroid hormone biosynthesis, estrogen signaling pathway, ovarian steroidogenesis, oxytocin signaling pathway, and folate biosynthesis. Notably, the levels of Oleic acid, Linoleic acid, L-Glutamine, Prostaglandin D2, 2-Hydroxyestradiol, and Estrone glucuronide were all higher in ResLac, whereas those of cAMP, Testosterone, and Folate were higher in ConLac (**Supplementary Table S6**).

### 3.7 Correlation analysis

**Figure 8:**
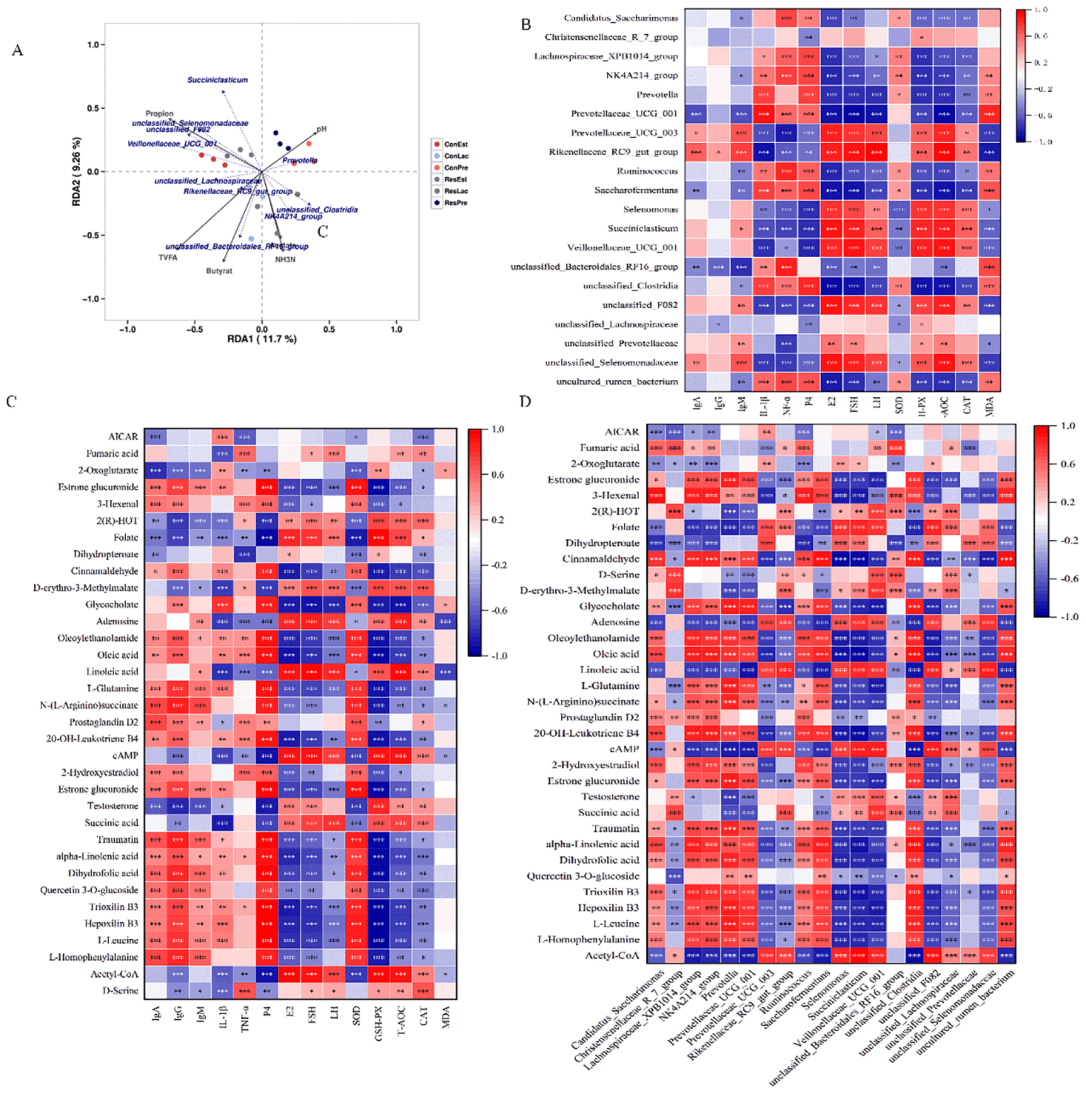
Correlation analysis among ruminal bacteria, differential metabolites, serum immunity, antioxidant and hormone levels. **(A)** RDA analysis of different microbiota and fermentation parameters; **(B)** Spearman’s correlation heatmap between the top 20 ruminal bacterial genera and serum indexes; **(C)** between the differential metabolites (from **Supplementary Table S2**) and serum indexes; **(D)** between the differential metabolites and top 20 ruminal bacterial genera. The color red and blue represent positive and negative correlations, respectively. The color intensity is proportional to the correlation values. *p < 0.05, **p < 0.01; ***p < 0.001.

Several metabolites (SCFAs, pH, NH_3_-N) were positively correlated with key microbial taxa (**Figure 8A**). Notably, *Rikenellaceae_RC9_gut_group* was positively correlated with TVFA and butyrate, and NH_3_-N was positively correlated with *NK4A214_group* (*p* < 0.05). Additionally, *Veillonellaceae_UCG_001* was positively correlated with propionate (*p* < 0.05).

The correlations between the top 20 ruminal bacterial genera and serum immunity, antioxidant, and hormone levels were analyzed using Spearman’s coefficient model. The findings revealed that Ig A and Ig M were negatively correlated with *Prevotellaceae_UCG_001*, but positively correlated with *Rikenellaceae_RC9_gut_group*, while IL-1β and TNF-α were positively correlated with *Prevotellaceae_UCG_001*, but negatively correlated with *Rikenellaceae_RC9_gut_group* (*p* < 0.001). Meanwhile, E2, FSH, LH, GSH-PX, T-AOC, and CAT were negatively correlated with *NK4A214_group*, *Prevotella*, *Prevotellaceae_UCG_001*, *Ruminococcus*, and *Saccharofermentans*, but positively correlated with *Rikenellaceae_RC9_gut_group*, *Succiniclasticum*, and *Veillonellaceae_UCG_001* (*p* < 0.01) (**Figure 8B**). Furthermore, Traumatin, alpha-Linolenic acid, 2-Hydroxyestradiol, L-Leucine, L-Homophenylalanine, L-Glutamine, Oleic acid, and Estrone glucuronide were positively correlated with Ig A, Ig G, P4, and SOD and negatively correlated with E2, FSH, GSH-PX, and T-AOC (*p *< 0.01). AICAR was negatively correlated with IgA, SOD, and CAT, but positively correlated with IL-1β (**Figure 8C**). Further analysis of the differential metabolites and top 20 ruminal bacterial genera revealed that metabolites such as Traumatin, alpha-Linolenic acid, L-Homophenylalanine, 2-Hydroxyestradiol, L-Glutamine, Oleic acid, and Estrone glucuronide were positively correlated with *Prevotella*, *Prevotellaceae_UCG_001*, *Ruminococcus*, *Saccharofermentans* and negatively correlated with *Succiniclasticum* and *Veillonellaceae_UCG_001* (*p *< 0.001) (**Figure 8D**).

### 3.8 Growth and development of newborn lambs and colostrum quality

**Figure 9:**
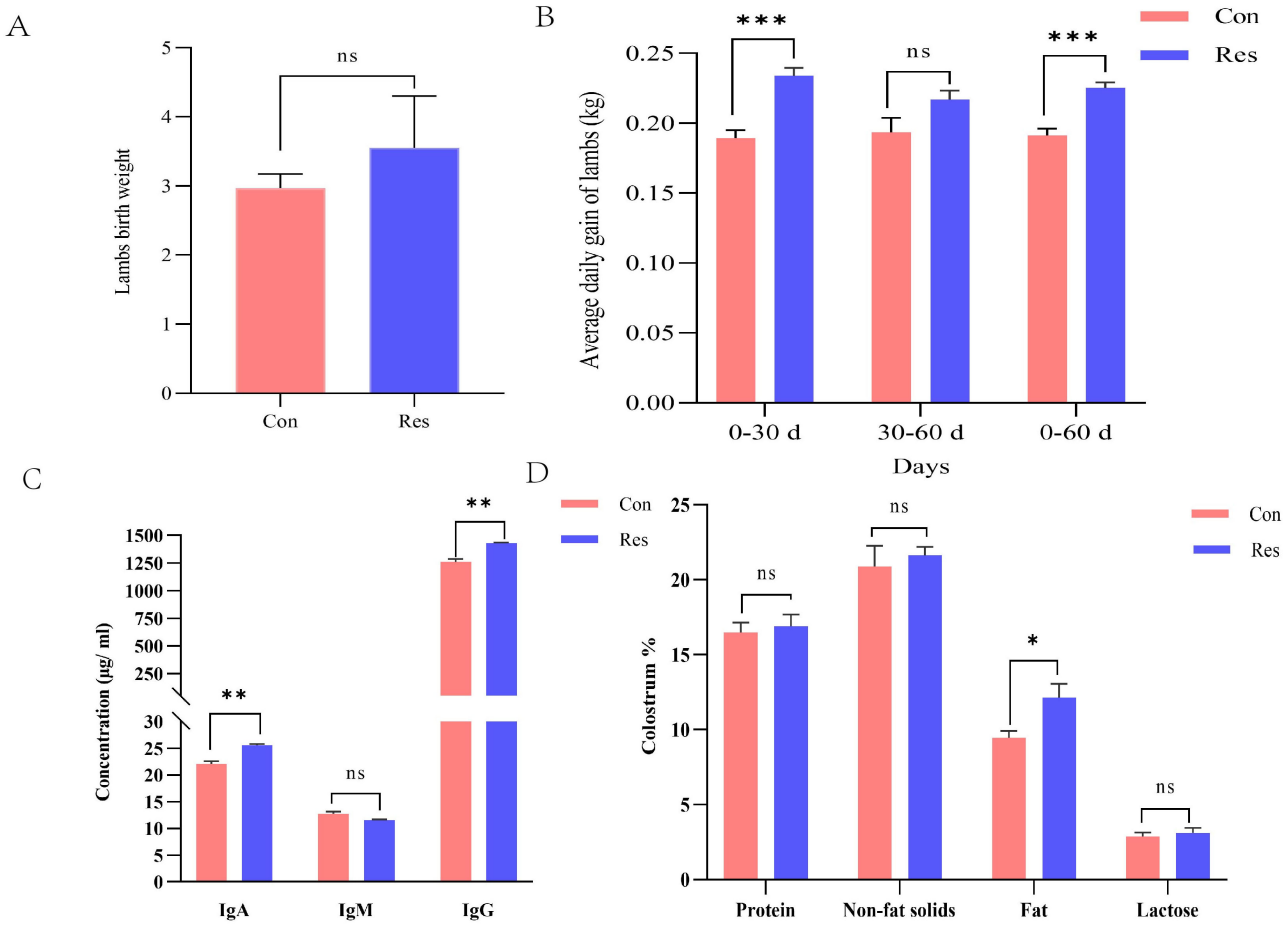
Effect of Res supplementation on weight of newborn lambs, average daily gain, and colostrum quality in ewes throughout the reproductive cycle. **(A)** Newborn weights*;* **(B)** Average daily weight gain of lambs; **(C)** Colostrum immunity indicators; **(D)** Colostrum qualities. *p < 0.05, **p < 0.01; ***p < 0.001.

Newborn weight tended to be higher (*p =* 0.097) and the average daily gain (ADG) was significantly higher (*p* < 0.001) following Res treatment in ewes (**Figures 9A, B**). Moreover, compared to the Con group, colostrum IgA (25.61 μg/mL), IgG (1429.10 μg/mL), and fat contents (12.14%) were significantly higher in the Res group (*p* < 0.05) (**Figures 9C, D**).

### 3.9 Res improves antioxidant activity and immunity by regulating rumen microorganisms and metabolites

**Figure 10:**
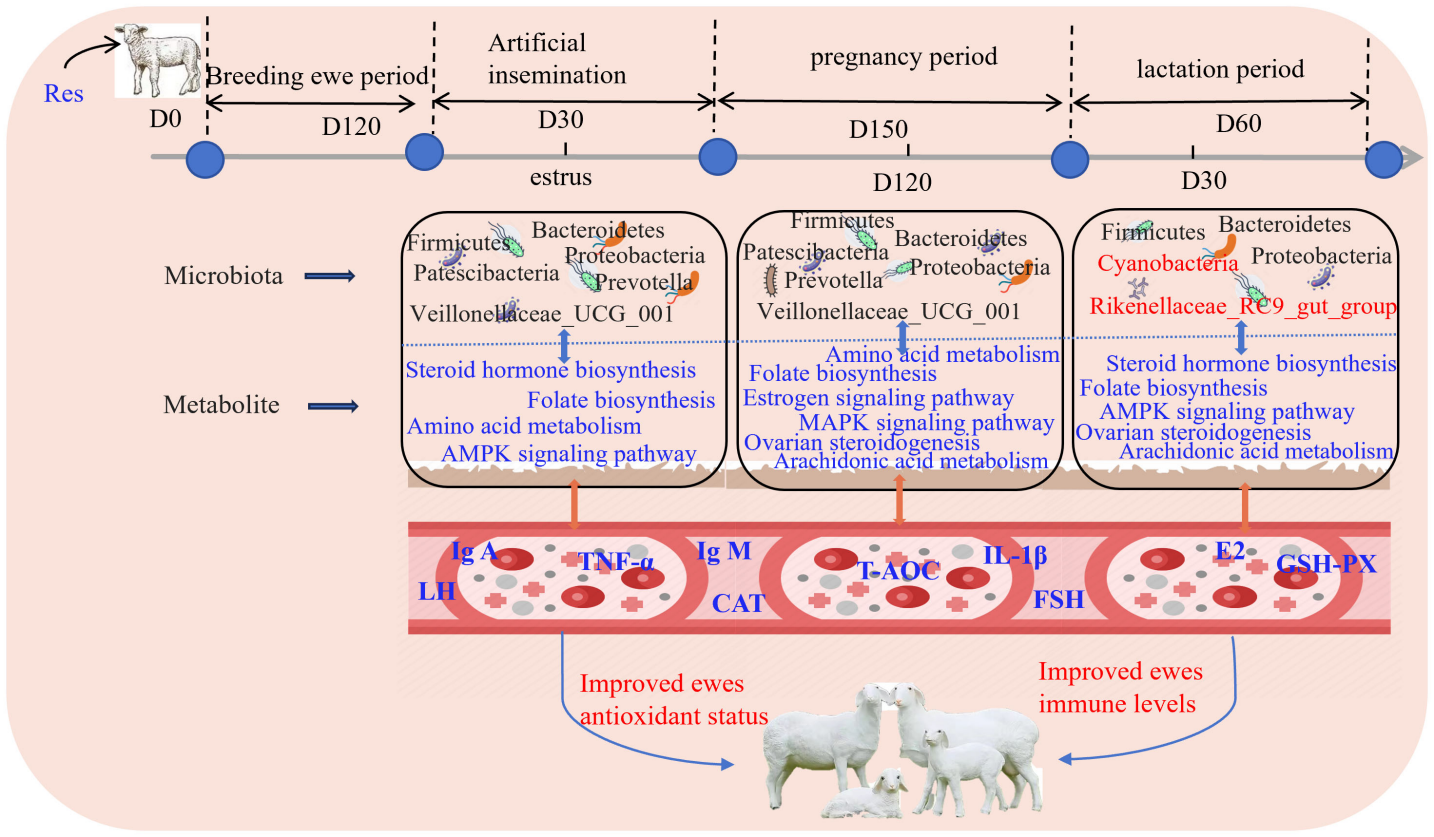
Feeding Res improves antioxidant performance and immunity of ewes.

Based on the results obtained in this study, a schematic illustration that depicts how Res supplementation improves antioxidant performance and immunity in ewes was generated (**Figure 10**). The findings revealed that Res altered the relative abundance of *Prevotella* and *Prevotellaceae_UCG_001* in the rumen during the pregnancy stages and increased the relative abundance of *Rikenellaceae_RC9_gut_group* during the lactation stage. These alterations led to changes in amino acid metabolism, the AMPK signaling pathway, folate biosynthesis, ovarian steroidogenesis, and other signaling pathways, in turn affecting the serum levels of GSH-Px, T-AOC, CAT, IgA, IgG, IgM, IL-1β, LH, P4, and FSH and thereby promoting antioxidant performance and immunity in ewes at all stages of reproduction.

## 4 Discussion

Intensive breeding makes ewes prone to oxidative stress, which can cause metabolic diseases, inflammation, and gastrointestinal dysbiosis. Previous studies have shown that Res exerts antioxidant, anti-inflammatory, and antibacterial effects in animals by regulating the composition of the intestinal microbiota (7). In this study, we investigated the effect of Res on antioxidant activity, immune responses, hormone levels, rumen microbiota and metabolites across various reproductive stages in ewes. The results showed that Res improved antioxidant activity and immunity in breeding ewes by regulating rumen microorganisms and metabolites, exerting beneficial effects on growth in offspring lambs. To our knowledge, this study is the first to examine the effects of continuous dietary Res supplementation on immunity and antioxidant performance in ewes throughout various reproductive stages.

Various intermediate metabolites in the blood, such as sugars, lipids, enzymes, proteins, etc. are closely related to the growth and development of animals, the level of body nutrition metabolism, physiological health, etc. When the animal body function is abnormal, the metabolic indicators in the blood will also change correspondingly. Alanine aminotransferase (ALT) and aspartate aminotransferase (AST) levels are commonly used to examine liver function, while cholesterol (CHO) and triglyceride (TG) levels reflect lipid metabolism and absorption (37). In this study, we found that Res significantly reduced the levels of ALT and AST in ewes during pregnancy and lactation, as well as the concentration of CHO during lactation. These findings were consistent with the results of Wu et al. in carp (*Cyprinus carpio*) (38), indicating that Res can improve metabolic health and liver function in ewes during pregnancy and lactation. In addition, glucose (GLU) Glucose is one of the important sources of energy for the animal body, and the GLU content in the blood can directly reflect the state of energy balance of the body, when the body lacks energy, the GLU content in the blood drops, we found increased GLU concentrations under Res treatment in ewes during lactation, which indicates that Res can improve energy levels in animals during this stage. Overall, these findings indicated that Res can improve health and potentially boost immunity in ewes.

Malondialdehyde (MDA) is a marker of oxidative stress, and total antioxidant capacity (T-AOC) is an objective index of the evaluate total antioxidant capacity of animals. Meanwhile, glutathione peroxidase (GSH-Px), catalase (CAT), and total antioxidant capacity (T-SOD) all have strong anti-free radical capacity (39). The negative energy balance commonly encountered by ewes during pregnancy and lactation leads to lipid mobilization and reactive oxygen species (ROS) production, which alter the immune and metabolic status of ewes and ultimately inducing oxidative stress (40). Res has attracted considerable attention owing to its unique antioxidant effects, which are mediated by the scavenging of free radicals and regulation of enzyme activity (41), In our study, we found that after Res treatment, GSH-Px and CAT activities were increased in ewes during lactation, T-AOC activity was increased during pregnancy and was accompanied by a reduction in MDA levels, and GSH-Px activity was increased during the estrus stage. These results indicated that adding Res to the diet could increase the activity of key enzymes involved in ROS scavenging, reduce MDA levels, and mitigate oxidative stress during pregnancy and lactation in ewes.

To further understand the effect of Res on immunity during pregnancy and lactation in ewes, immunoglobulins were examined to understand the status of humoral immunity (42). The results revealed that after Res supplementation, serum IgA, IgG, and IgM levels increased significantly during pregnancy and lactation, indicating that as an active polyphenol, Res could improve immunity levels in ewes (43). Res has previously been found to promote immune function and anti-inflammatory responses in animals (44). In this study, Res significantly reduced IL-1β concentrations during lactation, but it had no significant effect on TNF-α levels, consistent with the results reported by Meng et al. (25). Importantly, Res could improve the overall levels of immunity in ewes, although its effect on the inflammatory response in breeding ewes warrants further research. Furthermore, we found that serum hormones estradiol (E2), follicle-stimulating hormone (FSH), and luteinizing hormone (LH) levels were significantly higher in the Res group than in the Con group at sexual maturity, and Res increased progesterone (P4) and LH levels during lactation and FSH and LH levels during estrus. This indicated that Res could affect the hormone levels of ewes. Interestingly, previous studies have shown that changes in hormone levels can also alter immune function in animals (45).

Notably, altered levels of reproductive hormone levels and immune factors can alter the intestinal microbiota at different reproductive stages (46). Maintaining the dynamic balance of the gastrointestinal microbiota appears to be important for alleviating inflammation and improving intestinal health in animals (47). In this study, we found that Res altered the microbial diversity in the rumen of ewes, increasing the ACE and Chao 1 indexes during lactation. A higher microbial diversity indicates better rumen health and can help in physiologically preventing environmental stress (48). At the phylum level, after Res supplementation, *Firmicutes* and *Bacteroidetes* were the dominant phyla in ewes during three periods in our study, and the dominant phyla distribution was similar to results of Li et al. in ewes (4). We found that the relative abundance of *Firmicutes* decreased and *Bacteroidota* increased under the Res treatment in ewes during estrus, which could reduce fat ewes deposition, thereby accelerating material circulation and energy flow, and contributing to the maintenance of ewes health (49). In addition, the relative abundance of *Patescibacteria* in the rumen during estrus and lactation increased, while that of *Patescibacteria* and *Proteobacteria* during pregnancy decreased. *Patescibacteria* participates in various biological processes, including nutritional metabolism, antibacterial pathways, and immune responses, and it has been linked to SCFA production (50). At the genus level, *Ruminococcus* and *Succiniclasticum* belong to *Firmicutes*, which obtain nutrients mainly by decomposing fibers and promoting the fermentation of fibers into short-chain fatty acids (51, 52). In this study, adding Res to the diet of ewes had no significant effect on the relative abundance of *Ruminococcus* and *Succiniclasticum*, indicating that Res did not affect the fiber degradation ability of the animals. It has been suggested that *unclassified_Lachnospiraceae* may play a role in antioxidant and anti-inflammation effects (53). The relative abundance of *unclassified_Lachnospiraceae* was decreased significantly after Res treatment during pregnancy and was negatively correlated with IgG in this study, suggesting that the effect of Res on the immune performance of ewes may be related to *unclassified_Lachnospiraceae*. A study showed that reduced oxidative stress and inflammation are probably related to the adjusted and the *Christensensllaceae R-7 group* in sows supplemented with Res, and our study also found that the relative abundance of *Christensensllaceae R-7 group* was decreased under Res treatment during lactation stage. Interestingly, we found that the relative abundance of *Prevotella* during pregnancy and *Rikenellaceae_RC9_gut_group* during lactation increased significantly under Res supplementation. Researchers have discovered that *Prevotella* can effectively degrade hemicellulose and starch, promotes nutrient digestion and absorption, and is associated with propionate production (54). In line with these findings, we observed a significant increase in propionate levels during pregnancy in our study. These elevated levels of propionate could ensure sufficient energy supply to the ewes and favored fetal development during pregnancy. Meanwhile, *Rikenellaceae_RC9_gut_group* is involved in nutrient digestion and absorption and degrades carbohydrates into SCFAs, which are important for maintaining gastrointestinal epithelial cells and regulating immune responses (55). Some studies have also found that SCFAs not only maintain maternal homeostasis during pregnancy but also affect immune function (56). Our correlation analysis showed that *Rikenellaceae_RC9_gut_group* was positively correlated with Ig A, Ig M, E2, FSH, LH, GSH-PX, T-AOC, and CAT levels and negatively correlated with IL-1β and TNF-α levels. Collectively, these results indicated that the changes in reproductive hormones, antioxidant activity, and immune indexes after Res supplementation are closely related to variations in the abundance of *Rikenellaceae_RC9_gut_group*, thus affecting metabolite levels (SCFAs) during different maternal reproductive stages.

Microbes and their metabolites usually synergistically influence host physiological functions (57). Our date showed a significant correlation between rumen microorganisms and metabolites in ewes. AICAR, as an activator of the AMPK pathway, can enhance autophagy and reduce trophoblast migration, and it plays an important role in inflammatory responses. Meanwhile, 2-Hydroxyestradiol, as a metabolite of E2, has been shown to activate AMPK (58, 59). Some studies have demonstrated that Res exerts anti-inflammatory and antioxidant effects by regulating the AMPK signaling pathway (60, 61). Our research revealed that Res decreased AICAR and 2-Hydroxyestradiol levels in ewes and downregulated the AMPK signaling pathway during lactation. Furthermore, AICAR was positively correlated with IL-1β and negatively correlated with SOD and CAT, whereas 2-Hydroxyestradiol was positively correlated with IgA and IgG. Hence, we speculate that Res exerts anti-inflammatory and antioxidant effects in breeding ewes by regulating AICAR and 2-Hydroxyestradiol metabolites levels and thereby modulating the AMPK signaling pathway. In addition, Res has also been found to exert anti-inflammatory effects by regulating the MAPK signaling pathway (62, 63). In our study, although Res upregulated the MAPK signaling pathway during pregnancy, cAMP — a critical metabolite of this pathway — was negatively correlated with IL-1β and *Rikenellaceae_RC9_gut_group* and positively correlated with E2, FSH and LH levels. cAMP promotes ovarian tumor progression and was initially found to mediate the action of hormones in mammalian cells, including gonadotropin releasing hormone (GnRH), FSH and LH. The cAMP-dependent signal transduction pathway is triggered by the binding of FSH and LH to their respective G protein-coupled receptors (FSHR and LHR) (64, 65). In this study, Res was found to upregulate reproduction-related signaling pathways, such as progesterone-mediated oocyte maturation, estrogen signaling pathway, and ovarian steroidogenesis, as well as cAMP levels. Together, our results indicated that changes in metabolites such as AICAR and cAMP are closely related to the synthesis of reproductive hormones, and Res affects metabolism during maternal reproductive stages by regulating reproduction-related signaling pathways. Currently, similar studies on the effects of Res in sheep are lacking, and therefore, further research is required to validate our results. The limitation of this study is that all the results were obtained under identical feeding conditions, environment, region, and maternal conditions. The rumen microbiota of ruminants receives the influence of many factors such as breed, age, physiological condition, ration, additives and geographical location. The microbial communities observed in studies conducted in China may differ from those in other countries or farms due to differences in environmental and management factors. Ration and additive treatments had the most significant effect on rumen microbiota, and although the feeding effects of Res were investigated in this study, it did not consider the influence of breed and geographical location. Therefore the widespread use of Res across the country and the world requires further research.

Further,the maternal intestinal microbiome and the metabolites produced by these microbes during pregnancy and lactation play a crucial role in the healthy development of offspring (66). In this study, Res increased the birth weight and average daily gain (ADG) in offspring lambs, as well as the relative abundance of *Prevotella* in pregnant ewes. The increase in the relative abundance of *Prevotella* in ewes led to the production of more acetate, which provided more energy to the ewes and fetuses, thereby enhancing the birth weight and ADG of the offspring lambs. Resveratrol has been shown to affect not only the mother, but also crosses the placenta and directly affects the fetus during pregnancy (67). In addition, the vitality of newborn is associated with suckling colostrum, which is important for growth and development. During lactation, colostrum and milk provide lambs with nutritional and immunological resources that enhance the immunity and health of infants and newborns (68). In the present study, maternal Res supplementation can increase colostrum IgA and IgG levels, which further protects newborns, and promoting the rapid growth and development of lambs, consistent with the findings on sows by Zhao et al. (26). Colostrum immunoglobulins exert multiple protective effects in newborns (26). In summary, our results suggested that maternal Res supplementation could increase birth weight and ADG in lambs by enhancing colostrum immunoglobulin levels and improving the rumen microbiota.

## 5 Conclusions

The dietary supplementation of Res altered the structure of the rumen microbial community in ewes, increasing the relative abundance of *Prevotella* during pregnancy and that of *Rikenellaceae_RC9_gut_group* during lactation, but decreasing the relative abundance of *unclassified_Lachnospiraceae* and *Prevotellaceae_UCG_001* during pregnancy. Accordingly, Res increased propionate production in the rumen, providing additional energy to the mother and promoting fetal development during pregnancy. Our findings showed that Res can improve the quality of colostrum and enhance the growth of offspring lambs. Additionally, Res can upregulate amino acid metabolism, folate biosynthesis, and ovarian steroidogenesis and alter metabolites such as cAMP, AICAR, and 2-Hydroxyestradio, thereby improving antioxidant and immune indexes in ewes across different reproductive stages. Collectively, these results demonstrate that Res can be employed as a green feed additive to improve the health of ewes, especially under intensive breeding conditions.

## Data Availability

The datasets presented in this study can be found in online repositories. The names of the repository/repositories and accession number(s) can be found in the article/**Supplementary Material**.
