# Supplementary material for: Dietary resveratrol improves immunity and antioxidant defense in ewes by regulating the rumen microbiome and metabolome across different reproductive stages
Source: Front Immunol. 2024 Oct 11;15:1462805. doi: 10.3389/fimmu.2024.1462805 (PMC11502325; doi:10.3389/fimmu.2024.1462805)
Supplement: Supplementary file 1 [file Table1.docx]

Supplementary Material

**Supplementary Table S1** Composition and nutrient contents of the experimental diet (DM basis).

| Stage | | Pre-breeding  (3-5 months old) | Late breeding  (5-7 months old) | No-pregnancy- pre-pregnancy | Late pregnancy | Lactation |
| --- | --- | --- | --- | --- | --- | --- |
|  |  |  |  |  |  |  |
| Content(%) | Whole corn silage | 30 | 30 | 15 | 10 | 20 |
|  | Wheat straw | 30 | 20 | 25 | 25 | 15 |
|  | Alfalfa | 0 | 0 | 25 | 30 | 30 |
|  | Corn | 10 | 20 | 15 | 14 | 15 |
|  | Wheat bran | 3 | 4 | 3 | 4 | 4 |
|  | Bean pulp | 10 | 11 | 7 | 8 | 6 |
|  | Cottonseed meal | 6 | 5 | 2 | 1 | 2 |
|  | Flax cake | 5 | 4 | 2 | 2 | 2 |
|  | Baking soda | 0.5 | 0.5 | 0.5 | 0.5 | 0.5 |
|  | Dicalcium phosphate | 1 | 1 | 1 | 1 | 1 |
|  | Salt | 0.5 | 0.5 | 0.5 | 0.5 | 0.5 |
|  | 4% Premix ^a^ | 4 | 4 | 4 | 4 | 4 |
|  | Total | 100 | 100 | 100 | 100 | 100 |
| Nutrient | Dry matter, DM (%) | 74.63 | 74.09 | 83.35 | 86.32 | 80.31 |
|  | Metabolizable energy, ME (MJ/kg) | 8.87 | 9.54 | 8.92 | 8.89 | 9.18 |
|  | Crude Protein, CP (%) | 13.68 | 14.03 | 12.60 | 13.07 | 13.13 |
|  | Neutral detergent fiber, NDF (%) | 39.98 | 33.82 | 36.72 | 36.28 | 33.78 |
|  | Acid detergent fiber, ADF (%) | 26.91 | 22.60 | 22.93 | 25.29 | 22.50 |
|  | Calcium, Ca (%) | 0.91 | 0.94 | 1.019 | 1.07 | 1.08 |
|  | Phosphorus, P (%) | 0.61 | 0.61 | 0.572 | 0.535 | 0.6 |

^a^One kilogram of the premix contained the following: VA 120 000-400 000 IU, VD_3_ 50 000-150 000 IU, VE ≥500 IU, Fe 500-5 000 mg, Cu 0-1000 mg,

Mn 500-4 000 mg, Zn 700-6 000 mg, Co 6-50 mg, Se 5-20 mg. Digestible energy was calculated, and other values were measured.


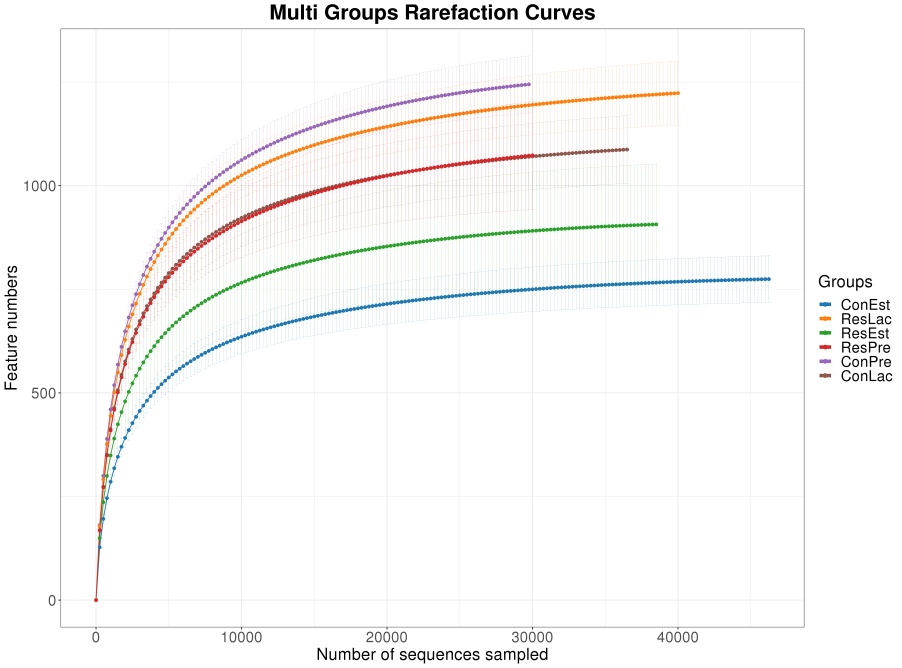

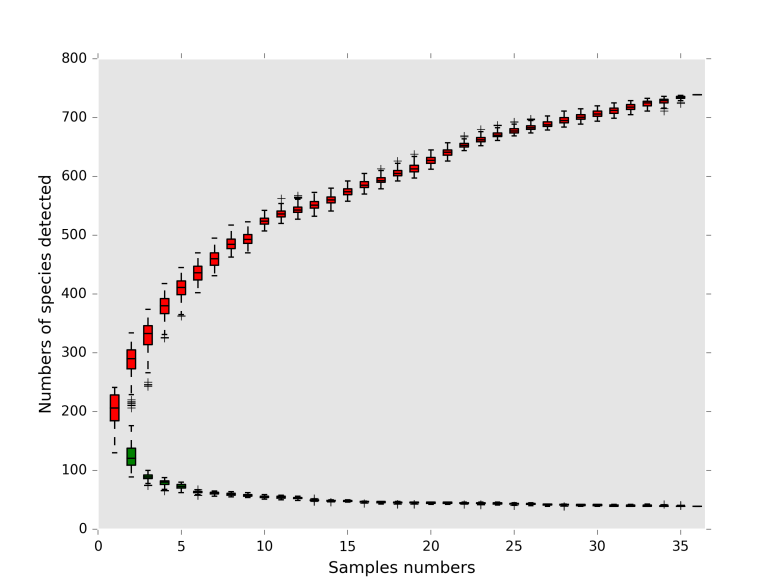


A

B

**Supplementary Figure S1.** Diversity analysis. **(A)** dilution curve analysis; **(B)** Species accumulation curve


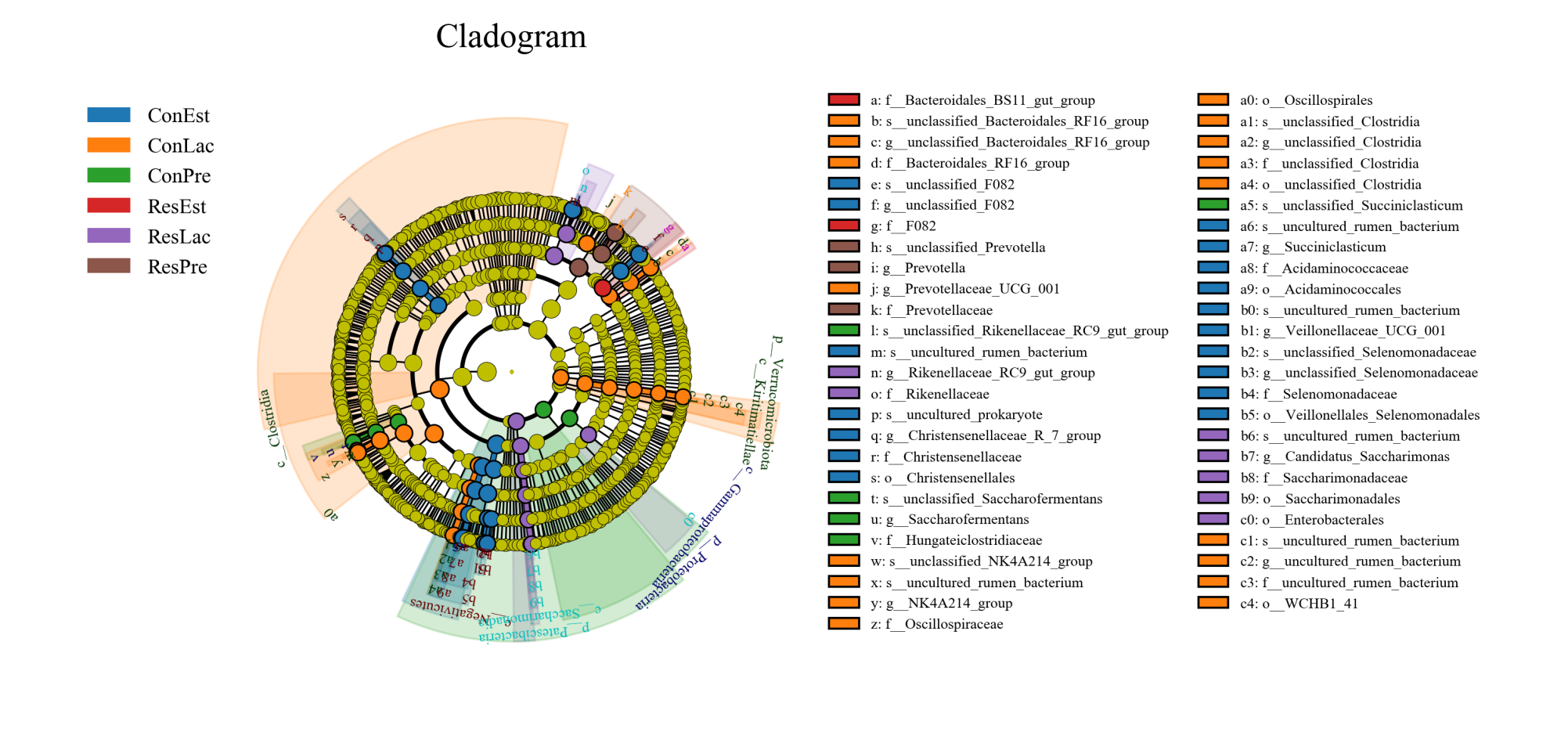


**Supplementary Figure S2.** LEfSe analysis. The linear discriminant analysis effect size (LEfSe) analysis of differential ruminal microorganisms in Con and Res group. Cladogram showed the significantly different bacteria from phylum to genus level. The nodes with different color represent the microbes that are significantly enriched in the corresponding groups and have a significant influence on the difference between the 2 groups. The yellow nodes represent the microbes that have no significant difference between the 2 groups.

ConPre vs ResPre

ConLac vs ResLac

ConEst vs ResEst


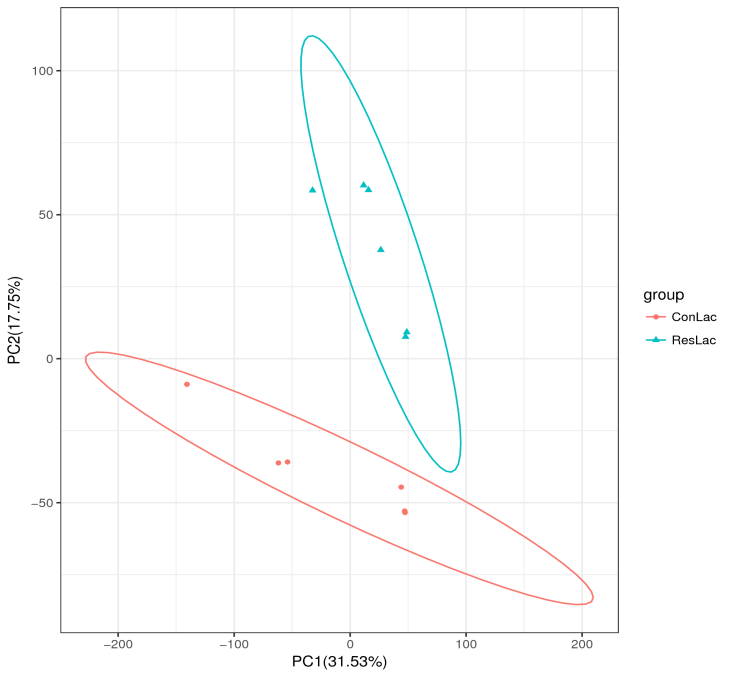

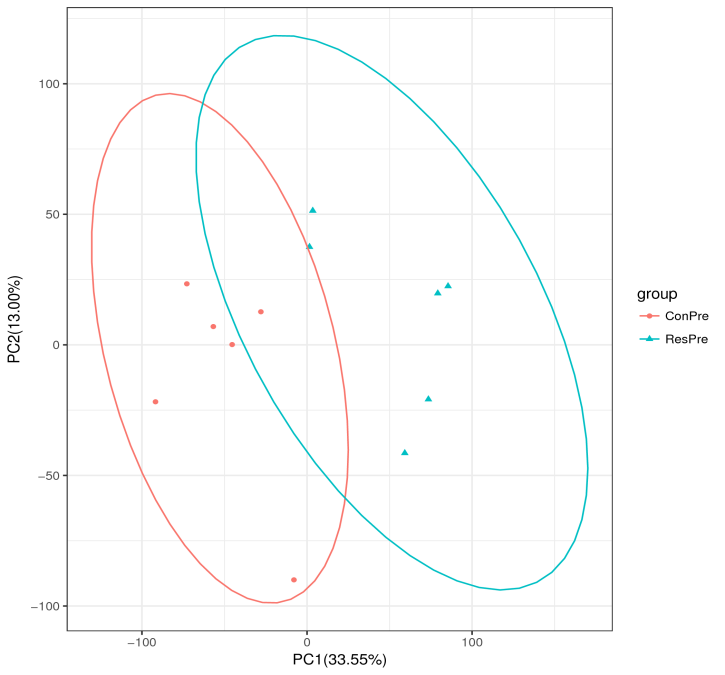

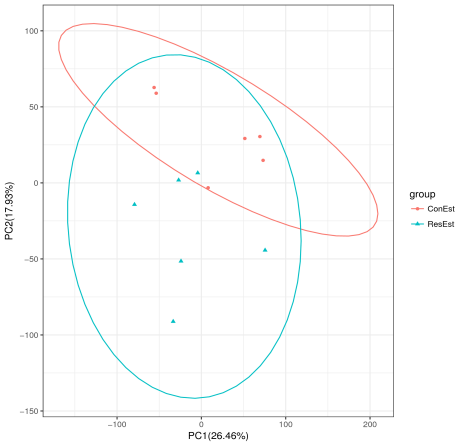


**Supplementary Figure S3.** Principal Component Analysis. ConEst = control in estrus; ResEst= resveratrol in estrus; ConPre = control in pregnancy; ResPre = resveratrol in pregnancy; ConLac = control in lactation; ResLac = resveratrol in lactation.


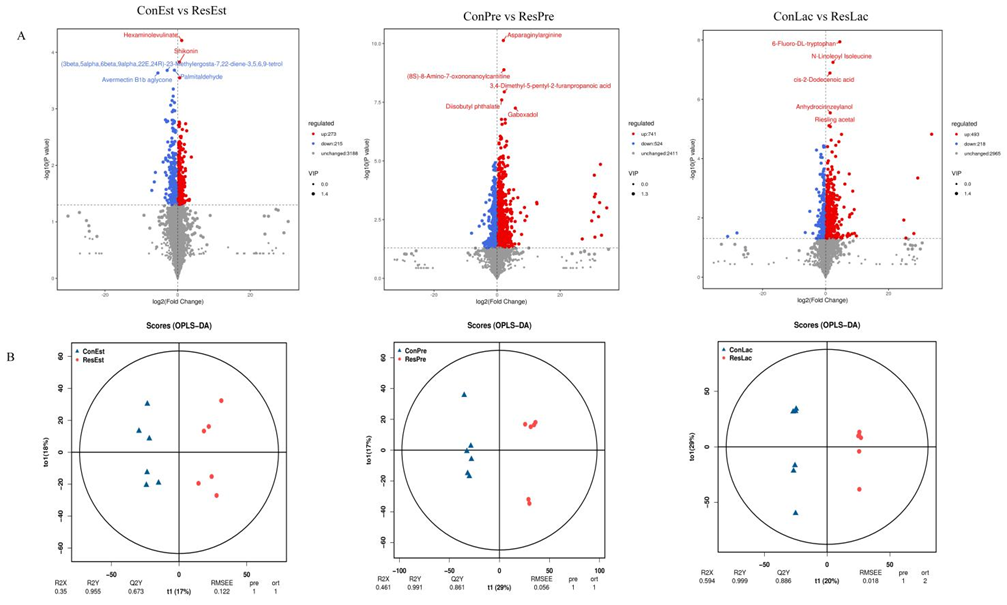


**Supplementary Figure S4.**Volcanic Maps and OPLS-DA Model Score Plots. **(A)** Volcanic maps of differential metabolites in three periods; **(B)** The OPLS-DA model score plots in three periods.

**Supplementary Table S6** Differential metabolic pathway enrichment analysis of significantly differential metabolites in the rumen.

| **Metabolic pathways** | **Metabolites** | ***p*-value** |
| --- | --- | --- |
| **ConEst vs.** **ResEst** | | |
| **Upregulation in the ResEst group** | **Upregulation/Downregulation in the ResEst group** |  |
| AMPK signaling pathway | AICAR | 0.009 |
| Steroid hormone biosynthesis | Estrone glucuronide | 0.004 |
| alpha-Linolenic acid metabolism | 3-Hexenal, 2(R)-HOT | 0.042/0.026 |
| Folate biosynthesis | Folate, Dihydropteroate | 0.002/0.044 |
| Inflammatory mediator regulation of TRP channels | Cinnamaldehyde | 0.033 |
| **Downregulation in the ResEst group** | **Upregulation/Downregulation in the ResEst group** |  |
| Biosynthesis of terpenoids and steroids | Fumaric acid, 2-Oxoglutarate | 0.048/0.049 |
| Amino acid metabolism (Glycine, serine and threonine, etc.) | D-Serine, D-erythro-3-Methylmalate | 0.000/0.034 |
| **ConPre vs.** **ResPre** | | |
| **Upregulation in the ResPre group** | **Upregulation in the ResPre group** |  |
| Insulin signaling pathway | cAMP | 0.001 |
| Oocyte meiosis | cAMP | 0.001 |
| MAPK signaling pathway | cAMP | 0.001 |
| Progesterone-mediated oocyte maturation | cAMP | 0.001 |
| Estrogen signaling pathway | cAMP | 0.001 |
| Ovarian steroidogenesis | cAMP | 0.001 |
| cAMP signaling pathway | Succinic acid | 0.040 |
| alpha-Linolenic acid metabolism | Traumatin, alpha-Linolenic acid | 0.017/0.039 |
| Folate biosynthesis | 7-Aminomethyl-7-carbaguanine, Dihydrofolic acid | 0.017/0.007 |
| Flavone and flavonol biosynthesis | Quercetin 3-O-glucoside | 0.002 |
| Arachidonic acid metabolism | Trioxilin B3, Hepoxilin B3, 5-HETE | 0.001/0.017/0.011 |
| Amino acid metabolism (Valine, leucine and isoleucine, etc.) | L-Leucine, L-Homophenylalanine, Acetyl-CoA | 0.001/0.003/0.022 |
| **ConLac vs.** **ResLac** | | |
| **Upregulation in the ResLac group** | **Upregulation/Downregulation in the ResLac group** |  |
| Cholesterol metabolism | Glycocholate | 0.028 |
| alpha-Linolenic acid metabolism | 13(S)-HPOT | 0.001 |
| cAMP signaling pathway | Adenosine, Oleoylethanolamide | 0.008/0.025 |
| Biosynthesis of unsaturated fatty acids | Oleic acid, Linoleic acid, gamma-Linolenic acid | 0.000/0.009/0.013 |
| Amino acid metabolism (Arginine, proline, Alanine, aspartate and glutamate, etc.) | L-Glutamine, N-(L-Arginino) succinate | 0.015/0.000 |
| Arachidonic acid metabolism | Prostaglandin D2, 20-OH-Leukotriene B4 | 0.043/0.003 |
| **Downregulation in the ResLac group** | **Upregulation/Downregulation in the ResLacgroup** |  |
| AMPK signaling pathway | AICAR | 0.000 |
| Oocyte meiosis | cAMP | 0.044 |
| Progesterone-mediated oocyte maturation |  |  |
| Estrogen signaling pathway |  |  |
| Steroid hormone biosynthesis | 2-Hydroxyestradiol, Estrone glucuronide | 0.027/0.009 |
| Ovarian steroidogenesis | 2-Hydroxyestradiol, Testosterone | 0.027/0. 020 |
| Folate biosynthesis | Folate | 0.001 |

ConEst = control in estrus; ResEst = resveratrol in estrus; ConPre = control in pregnancy; ResPre = resveratrol in pregnancy; ConLac = control in lactation; ResLac = resveratrol in lactation.
